# Modified F configuration in the treatment of Pauwels type III femoral neck fracture: a finite element analysis

**DOI:** 10.1186/s12891-021-04638-2

**Published:** 2021-09-06

**Authors:** Shiyuan Lin, Jie Shang, Baizhou Xing, Biao Wu, Rong Peng, Gang Wang, Hua-Ding Lu

**Affiliations:** 1 Department of Orthopaedics, The Fifth Affiliated Hospital of Sun Yat-Sen University, No. 52, Meihua East Road, Zhuhai, 519000 Guangdong Province China; 2 Department of Orthopaedics and Traumatology, Nanfang Hospital, Southern Medical University, Guangzhou, Guangdong China

**Keywords:** F-technique, Femoral neck fracture, Finite element analysis, Internal fixation, Pauwels type III

## Abstract

**Background:**

The optimal treatment of Pauwels type III femoral neck fracture (FNF) in young patients remains a worldwide challenge in orthopedic surgery.

**Methods:**

Finite element models of four internal fixations were developed to treat Pauwels type III FNF: a: the traditional inverted triangular parallel cannulated screw (PCS) model, b: the F-technique cannulated screw model, c: the modified F-technique cannulated screw model using a fully threaded screw instead of a partially threaded distally, d: the dynamic hip screw coupled with derotational screw (DHS + DS) model. Under the same conditions, finite element analyses were carried out to compare the displacement and von Mises stress distribution of four internal fixations and femurs, the maximum crack distances of the fracture surfaces, Z axis displacements of four models as well as the stress distribution in the subtrochanteric region.

**Results:**

The modified F-technique configuration resulted in a more stable fixation as compared to the other three configurations, with respect to the maximum displacement and stress peaks of femur and internal fixations, the maximum crack distances of the fracture surfaces, Z axis displacements of four configurations as well as the stress distribution in the subtrochanteric region.

**Conclusions:**

Our results suggested that modified F-technique configuration show a better performance in resisting shearing and rotational forces in treating Pauwels type III FNF compared to those using traditional inverted triangular PCS, the F-technique configuration or DHS + DS, providing a new choice for the treatment of FNFs.

## Introduction

Femoral neck fracture (FNF) tends to extend in a vertical fashion secondary to high-energy trauma in the young [1, 2]. According to the widely used Pauwels classification described in 1935, FNF line > 50 ° from the horizontal plane is Pauwels type III [3]. Internal fixation rather than arthroplasty is performed for nearly all FNFs in the young patients to preserve the native hip joint. Because of the high shearing force, type III FNF is considered as a troublesome injury with a high rate of complication including fixation failure, malunion, nonunion, and avascular necrosis [4–6].

At present, the available internal fixation methods for FNFs can be divided into three types [7, 8]: PC [9], DHS [10] and proximal femur plates [11]. A multicenter RCT study suggest that some groups of patients (smokers and those with displaced or base of neck fractures) might do better with a DHS than with PCS [12]. However, a meta-analysis reviewed that DHS had a higher rate of avascular necrosis than PCS [13]. A novel method of biplane double-supported F-technique screw fixation is capable of eliminating the shear stress and increased fixation strength [14, 15]. Moreover, the use of fully threaded PCS in traditional triangular configuration has a better performance in vertical FNFs biomechanically and get satisfied clinical outcome [16]. The study of Wegner et.al suggested that fully threaded cannulated screws have a better performance in resisting vertical shear force [17]. Then, if we make a combination of full threaded screw and F-technique configuration, we might get both of their benefits.

Thus, we hypothesized that replacing the distal partially threaded screw with a fully threaded screw in F-technique configuration might improve the stability of type III FNF. The purpose of this study is to introduce a modified F-technique configuration and evaluate its stability in fixing Pauwels type III FNFs by comparing with other three configurations biomechanically.

## Materials and methods

### Building a geometric model

A 32-year-old healthy male volunteer was recruited without the history of hip and systemic disease. Using a Toshiba Aquilion 64-row spiral CT scanner, a layer thickness of 1 mm CT scan of the femur was performed. The CT image was stored as a DICOM format and filed into the medical three-dimensional reconstruction software Mimics 16.0. A three-dimensional model of the femur was built based on the gray value of the tissue and segmentation of the region, and then exported as a stl format file [9]. This model was incorporated into Geomagic Studio 11 software for smoothing, meshing and fitting surface processing and incorporated into the Solidworks 2014 software (Dassault, France). The three-dimensional model of cortical bone and cancellous bone was developed by Boolean operation, and the femoral bone model was built re-assembly.

### Building the Pauwels III FNF model

We used Solidworks software to simulate the Pauwels type III unstable fracture. We first created the femoral shaft axis, a cross which a sagittal plane was created [9]. Then, we created a cutting plate that was across the center of the femoral neck at an angle of 20° with respect to the sagittal plane of the shaft axis [9]. The femoral neck was cut by the cutting plane, simulating a Pauwels type III fracture [9].

### Building the internal fixation model

#### Building the DHS + DS model

Cannulated screws with a diameter of 6.3 mm were fabricated using Solidworks software according to the clinical Synthes screw data. Because the focus of this study was related to the thread, the details of the thread were imitated. The thread portion was 16 cm in partly threaded cannulated screw. According to the literature reported [18, 19], the DHS was fabricated with Solidworks software.

#### Building the four internal fixation models

Building the traditional PCS model (a model): According to the surgical method reported in the literature [20], the screws were arranged in an inverted triangle parallel manner in the Solidworks software, and the angles of the three screws were 135° with the longitudinal axis of the femur.

Building the F-technique cannulated screws model (b model): according to the surgical method reported in the literature [21], the assembly model was built in Solidworks software with three partly threaded cannulated screws. The first one was placed into the distal screw. Taking the greater trochanter lower edge 6 cm as the entry point, and the axis of the screw and the diaphysis was 160°. The screw was placed from the anterior lower to the upper posterior to make it close to the femoral calcar. The second screw was placed in the middle, whose entry point was at the proximal end of the distal screw 3 cm. The screw was at an angle of 135 to the axis of the diaphysis. The third screw was placed 2 cm proximally from the entry point of the middle screw and parallel to it.

Building the modified F-technique cannulated screws model (c model): the distal partial-threaded cannulated screw is replaced by a full threaded screw in F-technique configuration.

Building the DHS + DS model (d model): the assembly model was built in Solidworks software according to the surgery method reported in literature [2, 19, 22]. The anti-rotational screw is parallel to the main screw of DHS.

**Fig. 1:**
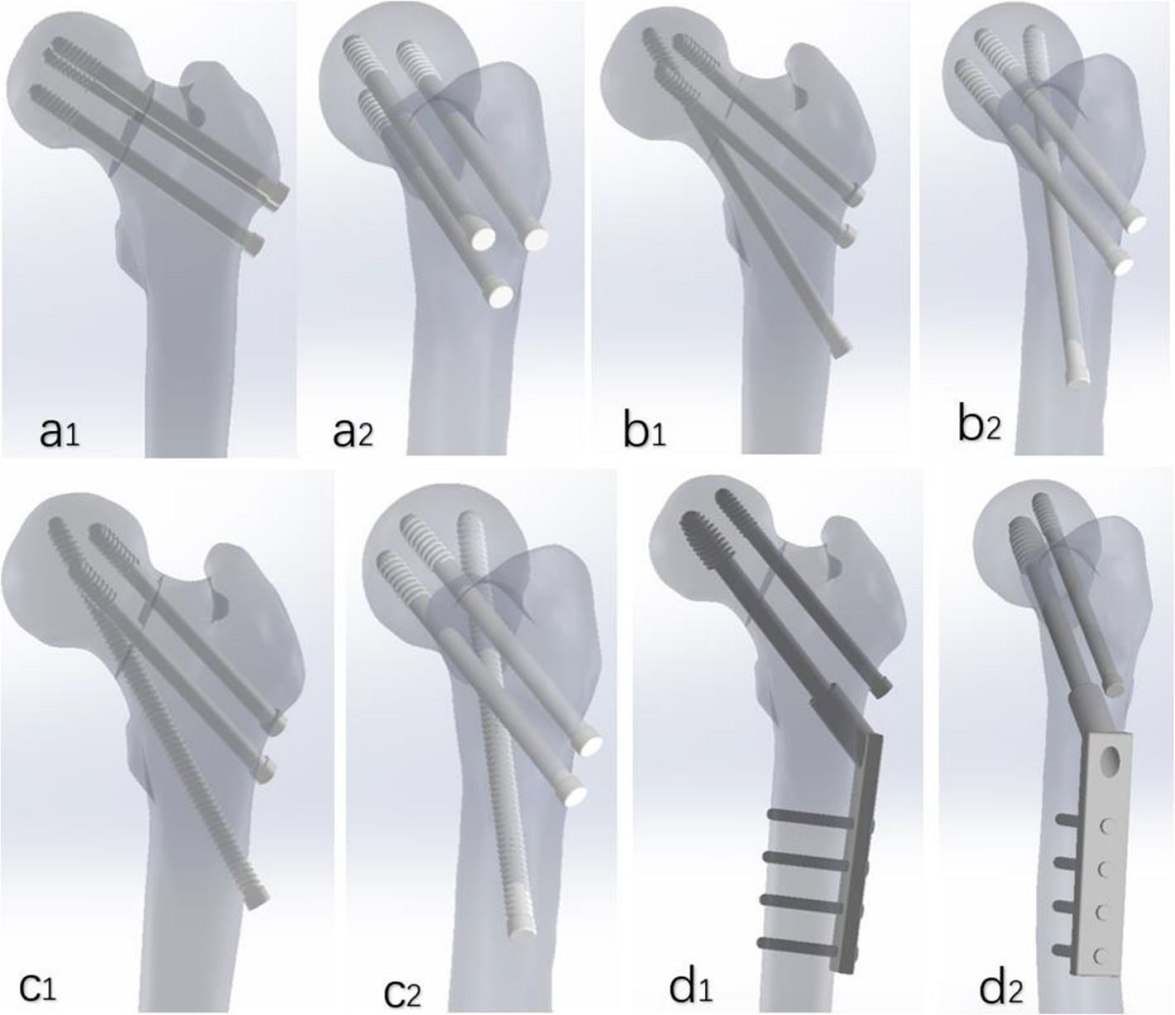
Geometric modeling of four internal fixation of femoral neck fracture. a1, a2: the traditional inverted triangular cannulated screw model; b1, b2: the F-technique cannulated screw model; c1, c2: the modified F-technique cannulated screw model using a fully threaded screw instead of a partially threaded distally; d1, d2: the dynamic hip screw coupled with anti-rotational screw model

Specific models are shown in Fig. 1. Subsequently, the models were incorporated into Ansys software (ANSYS company, America) for meshing and mechanical analysis.

### Conditional assumptions and material parameter settings

The fracture surface was completely fractured and was assumed that had been repositioned. The relationship between threaded surfaces of screws and bone was considered to be tie constraints. The interfaces between bone and partly thread screw body were set as friction, and the coefficient of friction factor was 0.3 [23]. Friction coefficients for bone-bone interaction was 0.46 [24]. The various materials in the model were assumed to be homogeneous, isotropic linear elastic materials. The screw was made of titanium alloy. The elastic modulus and Poisson’s ratio of various structural materials were shown in Table 1 [9].

**Table 1:** Bone and internal fixation material properties

Material	Elastic modulus (MPa)	Poisson’s ratio
Cortical bone	16,800	0.3
Cancellous bone	840	0.2
Titanium alloy	20,600	0.3

### Boundary conditions and loading force settings

All nodes on the surface of distal femur were constrained with 0 degrees of freedom to prevent rigid body motions during the analysis. This study simulated the forces loading on the hip during the stance phase of walking. The finite element models were applied a load (the force vector pointed laterally at an angle of 13° with the axis of the femoral shaft on the coronal plane, posteriorly by an angle of 8° with the shaft in the sagittal plane) of 2100 N corresponding to 300% body weight, and the force was introduced to the center of the femoral head [25].

### Evaluation criteria

First, the von Mises stress distributions and stress peaks of femur and four internal fixations were examined. Second, the maximum crack distances of the fracture surfaces as well as Z axis displacements of four configurations was measured. In this study, Z axis was defined by the projection of the direction of the load onto the fracture plane. Z axis represents the direction of the shear force. Last, the stress distribution in the subtrochanteric region in a, b and c model were measured.

## Results

### Displacement changes

**Fig. 2:**
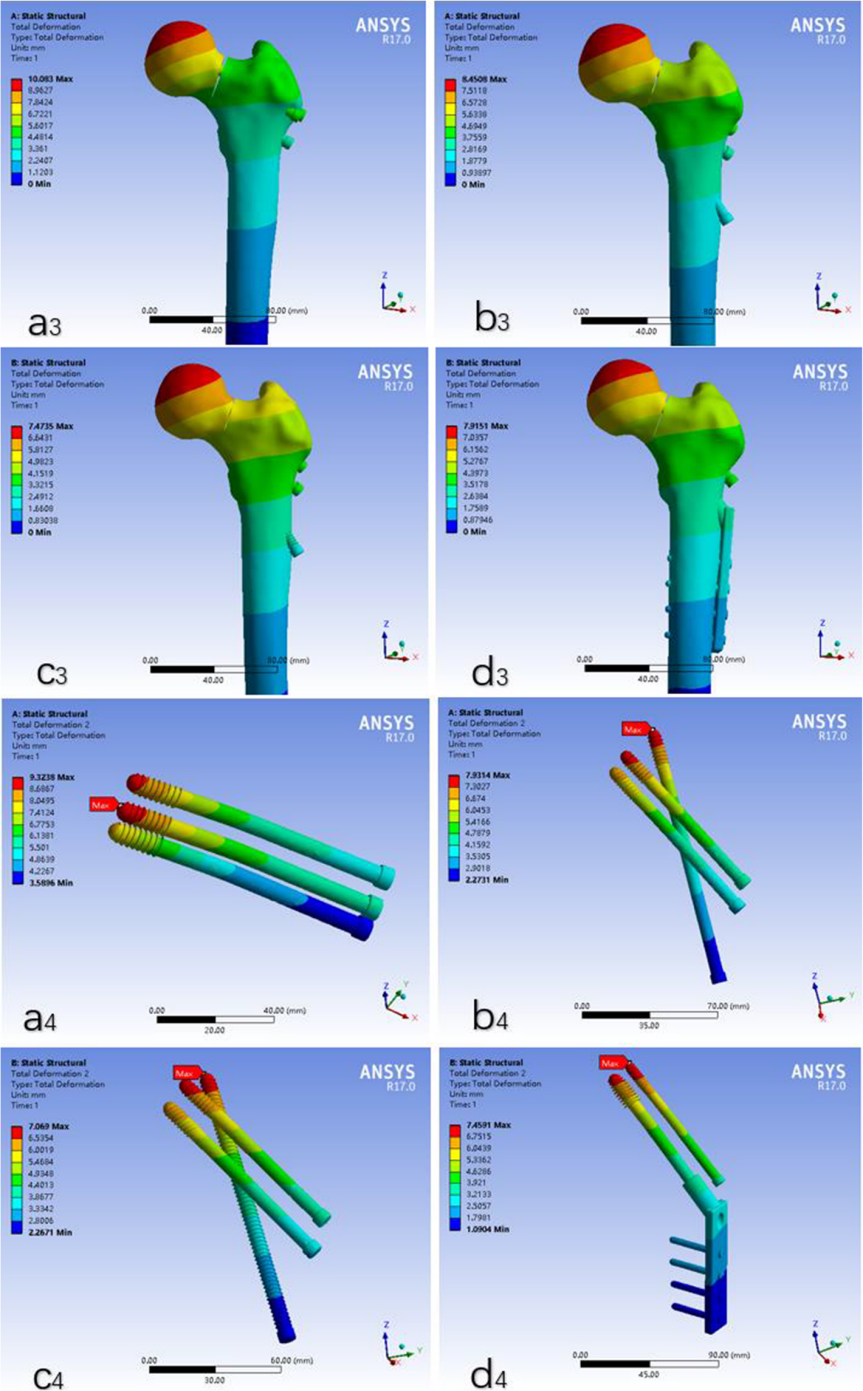
The displacement of femur and internal fixation in four models. a3, a4: the traditional inverted triangular cannulated screw model; b3, b4: the F-technique cannulated screw model; c3, c4: the modified F-technique cannulated screw model; d3, d4: the dynamic hip screw coupled with anti-rotational screw model

The number of elements and nodes of the models was listed in Table 2. The maximum femur displacement of the 4 models (a: the traditional inverted triangular PCS model, b: the F-technique cannulated screw model, c: the modified F-technique cannulated screw model, d: the DHS + DS model) were 10.083 mm, 8.4508 mm, 7.4735 mm and 7.9151 mm, respectively. (Fig. 2).

**Table 2:** The statistics of four assembly elements and the total amount of nodes

Case model	Nodes	Elements	Mesh size
a	318,251	202,045	1 mm
b	318,200	201,534	1 mm
c	344,398	216,206	1 mm
d	321,796	203,017	1 mm

a: the traditional inverted triangular cannulated screw model, b: the F-technique cannulated screw model, c: the modified F-technique cannulated screw model, d: the dynamic hip screw coupled with anti-rotational screw

The maximum internal fixation displacements were 9.3238 mm, 7.9314 mm, 7.0690 mm and 7.4591 mm in a, b, c and d models, respectively. (Fig. 2).

The maximum crack distances of the fracture surfaces of the 4 internal fixation models a, b, c and d were 0.53 mm, 0.63 mm, 0.41 mm and 0.47 mm, respectively (Fig. 2). The maximum crack distances of the fracture surfaces in modified F-technique configuration was the smallest of the four models. The crack distances of the fracture surfaces represent the ability to resist varus of femoral head.

**Fig. 3:**
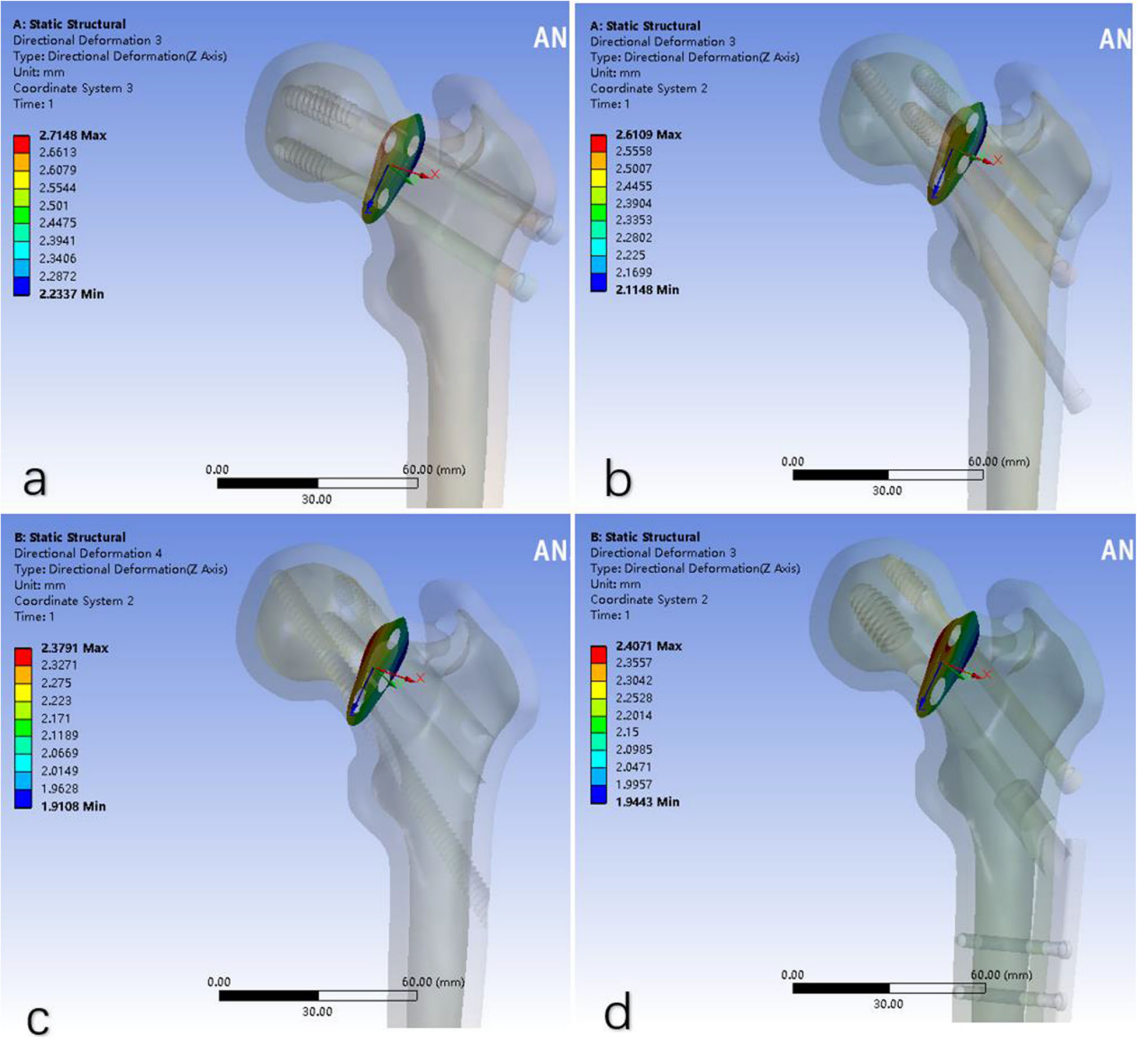
The displacement of Z axis in four models (it represents the shear force direction)

The maximum Z axis displacements of the 4 internal fixation models a, b, c and d were 2.7148 mm, 2.6109, mm 2.3791 mm and 2.4071 mm, respectively (Fig. 3). As we can see, the c model has the minimum results, demonstrating that the modified F-technique configuration exhibited less displacement than did the other three models. Z axis represents the shear force direction.

### The Von Mises peak stress distribution

**Fig. 4:**
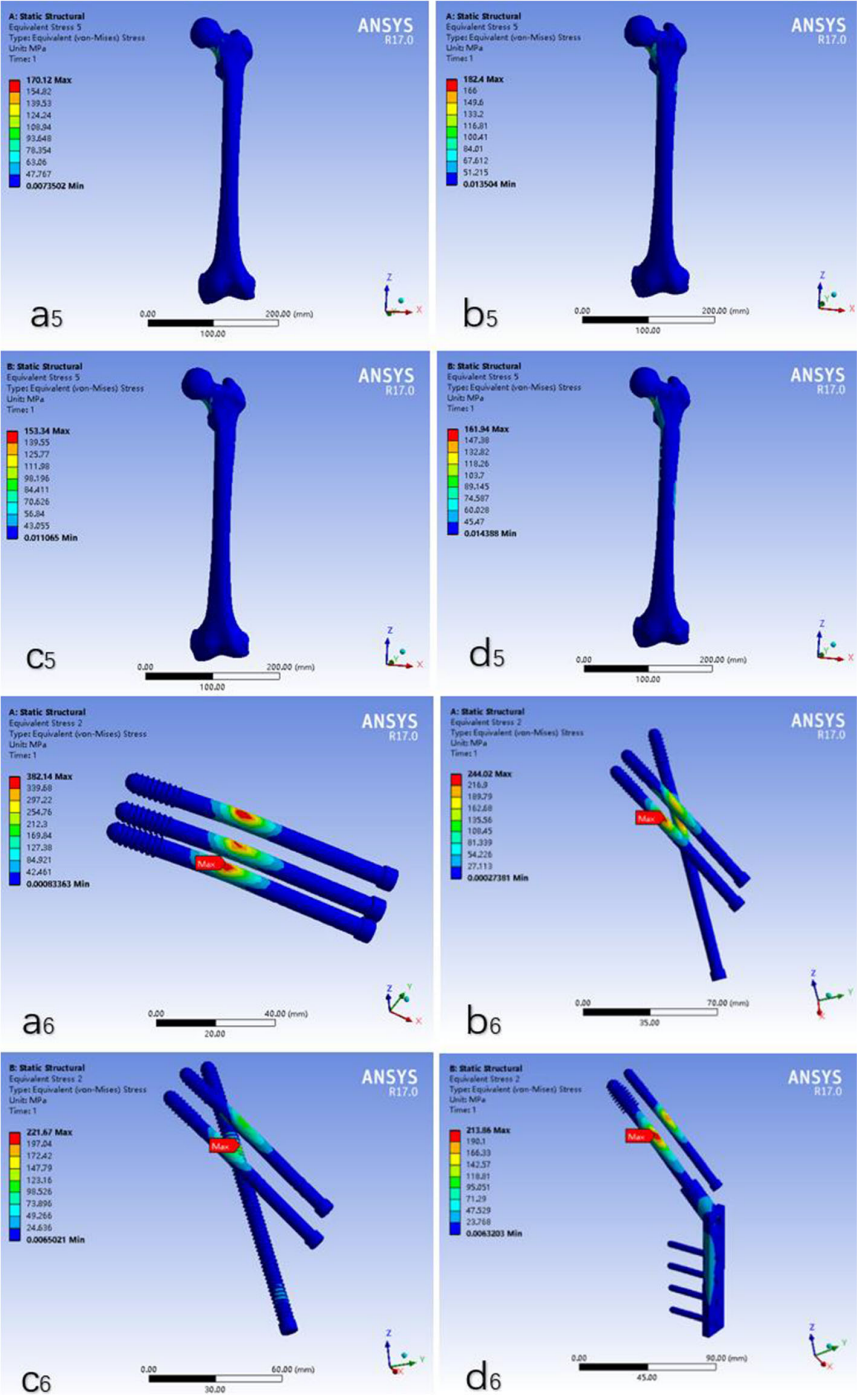
The stress of the femur and internal fixation in four models. a5, a6: the traditional inverted triangular cannulated screw model; b5, b6: the F-technique cannulated screw model; c5, c6: the modified F-technique cannulated screw model; d5, d6: the dynamic hip screw coupled with anti-rotational screw model

Differences of stress distribution were observed on the four configurations and the femur. The peak Von Mises stresses at the femoral head were 170.12 MPa, 182.40 MPa, 153.34 MPa and 161.94 MPa in a, b, c and d models, respectively (Fig. 4). The peak Von Mises stresses of internal fixation were 382.14 MPa, 244.02 MPa, 221.67 MPa and 213.86 MPa in a, b, c and d models, respectively (Fig. 4).

**Fig. 5:**
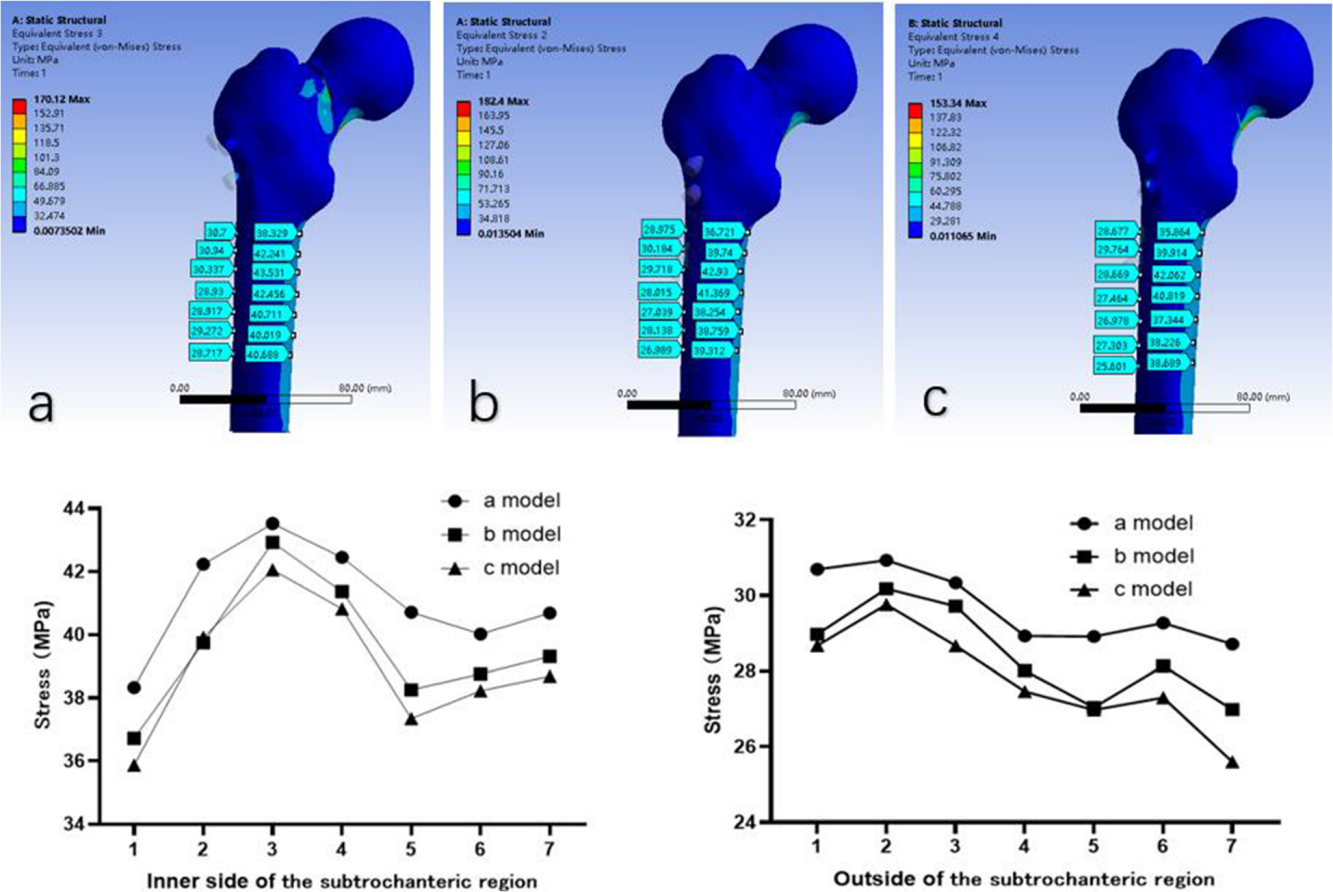
The stress distribution below the lesser trochanter in the subtrochanteric region of the femur in three models. a: the traditional inverted triangular cannulated screw model; b: the F-technique cannulated screw model; c: the modified F-technique cannulated screw model

The stresses distribution in the subtrochanteric region of a, b and c model were showed in Fig. 5. The largest stress was observed in both outside and inner side of the subtrochanteric region in a model.

Table 3 shows the results in detail.

**Table 3:** Parameters results

Parameters	a	b	c	d
The maximum displacement of the femur (mm)	10.083	8.4508	7.4735	7.9151
The maximum displacement of the internal fixation (mm)	9.3238	7.9314	7.069	7.4591
Maximum femur stress (MPa)	170.12	182.4	153.34	161.94
Internal fixation maximum stress (MPa)	382.14	244.02	221.67	213.86
The maximum crack distances of the fracture surfaces (mm)	2.4625	1.5006	0.793	1.278
The maximum displacement of Z axis (mm)	2.7148	2.6109	2.3791	2.4071

a: the traditional inverted triangular cannulated screw model, b: the F-technique cannulated screw model, c: the modified F-technique cannulated screw model using a fully threaded screw instead of a partially threaded distally, d: the dynamic hip screw coupled with anti-rotational screw

## Discussion

The results of this study show that compared with the other three configurations, the modified F-technique screw fixation configuration are the smallest with respect to the maximum femoral and internal fixation displacement, the maximum displacement in the direction of shear force, the maximum crack distances of the fracture surfaces and the maximum femoral stress, indicating that the performance of modified F structure in anti-shearing force and anti-varus of femoral head is stronger than that of DHS + DS model, F-technique screw model and inverted triangle PCS model. Therefore, the modified F-technique configuration fixes Pauwels type III FNFs getting the best stability performance. When encountering Pauwels type III FNFs that require internal fixation in practice, the modified F-technique screw fixation configuration can be considered first, followed by DHS + DS and F screw fixation.

Pauwels type III FNFs usually occur in young patients with high-energy injuries [2, 26]. In such patients, hip-preserving treatment with reduction and internal fixation is usually the gold standard [7]. In Pauwels type III FNFs, because the fracture line is more inclined, the shear force is dominant, and the varus force is large, which easily leads to fracture displacement and varus collapse [27]. The existence of these two forces must be fully considered when choosing internal fixation [7, 27]. It has been reported in the literature that the nonunion rate of Pauwels type III FNFs is 16–59%, and the incidence of femoral head necrosis is 11–86% [28]. Poor prognosis such as femoral head necrosis and fracture nonunion will bring catastrophic consequences to patients. However, at present, the best treatment for Pauwels III FNFs is still inconclusive, exploring the best fixation methods for FNFs is one of the current research focuses [28].

In order to overcome the greater shearing force of Pauwels type III FNFs, some authors have proposed a treatment plan that uses screws and medial femoral neck plates. Related mechanical studies have shown [15, 29] that the medial femoral neck plate can effectively resist shear forces. However, it is difficult to install a steel plate on the inner of the femoral neck because the femoral neck is deep and the area is close to the joint capsule of the hip joint. Placing the steel plate will inevitably destroy the joint capsule structure. It may also damage the blood supply of the femoral head, especially the lower retinaculum artery, which is considered to play an important role in the blood supply of the femoral head after a FNF [30]. The need to increase the medial steel plate is bound to increase an incision, and the operation time and trauma will be greater. Therefore, the model of the medial femoral neck plate was not used in this study.

PCSs have been used for the treatment of FNFs for many years. They have the advantages of minimal invasion, low blood loss, short hospital stay, and short operation time [31, 32]. However, among the four fixation models in this study, the three parallel screws of the inverted triangle have the weakest resistance to shear force and the varus of the femoral head when fixing the Pauwels type III FNFs. So, this configuration is not recommended to be used in fixing Pauwels type III FNFs. Research by Gurusamy et al. showed that within the first 3 months after surgery, the failure rate of 3 PCSs was as high as 39% [33]. Research by Liporace et al. also showed [28] that the nonunion rate of Pauwels type III FNFs fixed with 3 PCSs was 19%. Stankewich et al. concluded in their cadaveric study that the use of PCSs to fix Pauwels type III FNFs is not recommended [34].

Our study showed that the shear resistance and anti-varus stability of the F-technique screw configuration is weaker than that of the DHS + DS model, but stronger than the PCS configuration. Filipov initially proposed to fix osteoporotic FNFs with the F screw fixation technique with dual-plane and dual-support [26]. Clinical studies have shown that the failure rate of this configuration in the treatment of FNFs is only 3.4% [21]. The finite element study of Lin et al. using F screw fixation configuration to treat Pauwels type III fractures showed that F screw fixation has higher stability than PCS fixation [15]. Our results are consistent with the conclusions of this study. However, the stability of F screw fixation is still lower than that of the DHS + DS fixation configuration.

In our study, the modified F configuration does not require additional incisions, and its shear resistance and anti-varus stability is the strongest among the four models. The distal screw adopts a full-thread setting, and the contact area between the bone and the screw is larger, so that it can obtain better shear resistance and mechanical stability. What is more, full-threaded screw provides better support rather than compression ability and can support the force transmitted from the femoral head. Therefore, the modified F configuration can obtain better early mechanical stability of FNF fixation. The biomechanical study of Zhang et al. [35] showed that the larger the Pauwels angle of FNFs, the more obvious the advantages of full-threaded screws to fix FNFs. Improving the fixation stability of FNF can improve the blood supply of femoral head and reduce the poor prognosis such as fracture nonunion and femoral head necrosis, which is the key factor for the fixation of unstable FNF [36]. The modified F configuration only replaces the distal half-threaded screw with a full-threaded screw. The upper two half-threaded screws still have a partial sliding pressure effect [25, 37–39]. The distal full-threaded screws can effectively prevent the femoral neck from shortening, because full-threaded screw mainly provides support rather than compression force. The modified F configuration provides a new ideal option for the treatment of Pauwels type III FNF.

Moreover, stresses distribution in the subtrochanteric region of modified F configuration was lower than that in a and b models, indicating that modified F configuration may not increase the risk of subtrochanteric fractures. The small distance of less than 7 mm between the three PCSs, used in traditional inverted triangular cannulated screw configuration, may be a significant stress-riser in subtrochanteric region. However, the rather wide distance between screws in F configuration (20–40 mm) might not weaken the subtrochanteric femur bone, because the tensile forces acting on the lateral cortex are spread over a larger area [21]. Filipov et.al conducted a study containing 207 FNFs treated with F configuration, he did not observe any iatrogenic subtrochanteric fracture after surgery [21].

The novelty of this study is that we propose a modify F configuration with strong stability in fixing Pauwels type III FNF. Moreover, finite element analyses were used with the primary goal of comparing various fixation methods by measuring the interfragmentary motions to assess more important parameters that are difficult and almost unlikely to experimentally measure, for example, the stress or strain distribution within the bone and implant [40]. The limitations of this study include simplifying the fracture model and internal fixation details as well as ignoring the effects of surrounding muscles and ligaments on fracture stability. The study is only a biomechanical analysis, and a randomized controlled trial is needed to further verify our results.

Through the present finite element analyses, it was found that the modified F cannulated screw technique is capable of eliminating the varus stress and shear stress while maintaining the axial compressive stress at the fracture end. Thus, this configuration can create a good mechanical environment for fracture healing. Accordingly, for the Pauwels III FNF, the use of modified F configuration is recommended, followed by DHS + DS, F screw fixation. However, PCS configuration is not recommended to be used in Pauwels type III FNFs.

### Statement

All the authors confirm that all experimental protocols were approved by the Ethics Committee of The Fifth Affiliated Hospital of Sun Yat-Sen University, affiliated to Sun Yat-Sen University.

All the authors confirm that all methods were carried out in accordance with relevant guidelines and regulations.

All the authors confirm that informed consent was obtained from the participant.

## Data Availability

The datasets generated during and analyzed during the current study are not publicly available due to the participate not agree to publish the raw data online but are available from the corresponding author on reasonable request.
